# Antimicrobial activity of lipids extracted from *Hermetia illucens* reared on different substrates

**DOI:** 10.1007/s00253-024-13005-9

**Published:** 2024-01-23

**Authors:** Antonio Franco, Carmen Scieuzo, Rosanna Salvia, Valentina Pucciarelli, Luca Borrelli, Nicola Francesco Addeo, Fulvia Bovera, Ambrogio Laginestra, Eric Schmitt, Patrizia Falabella

**Affiliations:** 1 Department of Sciences, University of Basilicata, Via Dell’Ateneo Lucano 10, 85100 Potenza, Italy; 2 Spinoff XFlies S.R.L, University of Basilicata, Via Dell’Ateneo Lucano 10, 85100 Potenza, Italy; 3 Department of Veterinary Medicine and Animal Production, University of Naples Federico II, Via F. Delpino 1, 80137 Naples, Italy; 4 Department of Relations With the Territory, TotalEnergies EP Italia S.P.A, Via Della Tecnica, 4, 85100 Potenza, Italy; 5 Protix B.V., Industriestaat 3, NC 5107 Dongen, The Netherlands

**Keywords:** Fatty acids, Antimicrobial resistance, Black soldier fly, Insects oil

## Abstract

**Abstract:**

As the problem of antimicrobial resistance is constantly increasing, there is a renewed interest in antimicrobial products derived from natural sources, particularly obtained from innovative and eco-friendly materials. Insect lipids, due to their fatty acid composition, can be classified as natural antimicrobial compounds. In order to assess the antibacterial efficacy of *Hermetia illucens* lipids, we extracted this component from the larval stage, fed on different substrates and we characterized it. Moreover, we analyzed the fatty acid composition of the feeding substrate, to determine if and how it could affect the antimicrobial activity of the lipid component. The antimicrobial activity was evaluated against Gram-positive *Micrococcus flavus* and Gram-negative bacteria *Escherichia coli*. Analyzing the fatty acid profiles of larval lipids that showed activity against the two bacterial strains, we detected significant differences for C4:0, C10:0, C16:1, C18:3 n3 (ALA), and C20:1. The strongest antimicrobial activity was verified against *Micrococcus flavus* by lipids extracted from larvae reared on strawberry, tangerine, and fresh manure substrates, with growth inhibition zones ranged from 1.38 to 1.51 mm, while only the rearing on manure showed the effect against *Escherichia coli*. Notably, the fatty acid profile of *H. illucens* seems to not be really influenced by the substrate fatty acid profile, except for C18:0 and C18:2 CIS n6 (LA). This implies that other factors, such as the rearing conditions, larval development stages, and other nutrients such as carbohydrates, affect the amount of fatty acids in insects.

**Key points:**

• *Feeding substrates influence larval lipids and fatty acids (FA)*

• *Generally, there is no direct correlation between substrate FAs and the same larvae FAs*

• *Specific FAs influence more the antimicrobial effect of BSF lipids*

**Graphical abstract:**

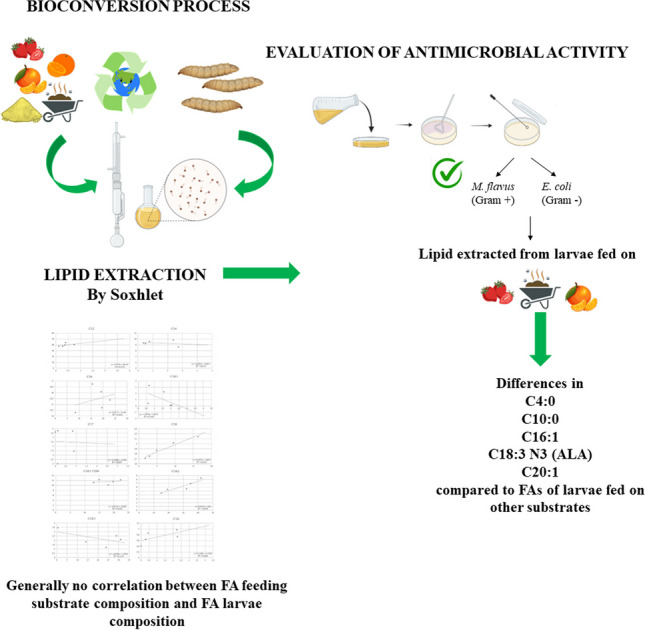

## Introduction

Antimicrobial agents are frequently misused in the treatment and prevention of diseases in human, animal breeding, and agricultural cultivation (Rodgers and Furones 2009). In recent decades, this misuse favored the selection of multi-resistant bacterial strains and allowed their spread through thoughtless actions. As a result, there is currently an urgent need to identify new antimicrobials of natural origin that could be also innovative and sustainable in terms of environmental safety and economic costs. Insects can be a good source of new antimicrobial compounds, not only for animal feed (http://www.fao.org/antimicrobial-resistance/key-sectors/animal-production/en/) but also for other fields, such as cosmetics (Franco et al. 2021; 2022a). Black soldier fly (BSF, *Hermetia illucens* (L.); Diptera: Stratiomyidae) is one of eight insects identified by the European Food Safety Authority (EFSA), having the greatest potential for use as feed in the European Union (EFSA 2015) and was used for different purposes, including the conversion of organic matters, even in decomposition (Diener et al. 2011, Scala et al. 2020; Scieuzo et al. 2022), the replacement of conventional protein sources in animal feed (Makkar et al. 2014; Maurer et al. 2015; Bovera et al. 2018), and the disposal of different substrates (for example, livestock manure and restaurant waste) (Spranghers et al. 2017; Franco et al. 2022b). Massive breeding of bioconverter insects as feed and/or food source has been a hot topic in recent years, with both economic and scientific aspects associated with rearing and subsequent processing optimization (Scala et al. 2020). BSF larvae (BSFL) can be exploited to produce proteins and lipids with high biological and economic value in an environmentally sustainable way (Wang and Shelomi 2017). In addition, BSFL are known to be a source of antimicrobial peptides (AMPs) that act as effective inhibitory compounds against a wide range of pathogens, whose expression can be modulated by different diet administration (Brown et al. 2008; Vogel et al. 2018; Manniello et al. 2021; Moretta et al. 2020; 2021; Di Somma et al. 2022). Antimicrobial compounds of BSFL are in the crude fat, too. Antimicrobial lipids, particularly single-chain amphiphilic lipids that potentially damage bacterial cell membranes, are promising candidates for future generation antimicrobial agents in the treatment of bacterial infections (Marusich et al. 2020). Their ability to destabilize the bacterial cell membrane makes them promising candidates among novel antimicrobials because these bacteria are also unlikely to acquire resistance to these compounds (Borrelli et al. 2021). With the ever-growing antimicrobial resistance, finding new candidates for antimicrobial drug development is indispensable. This study contributes to strengthen not only the evidence about the antimicrobial properties of lipids extracted from BSFL, but also the characteristics of the substrates to be used to enhance these properties. Through its bioconversion processes, BSF produces different amounts of crude fat, which are present in high quantities in mature larvae and prepupae, depending on the feed substrates (Franco et al. 2021). Lipids can be used as animal feed ingredients, in the formulation of numerous products for personal care (Franco et al. 2021, 2022a) and for biodiesel production (Zheng et al. 2012). The composition of insect lipids is determined by the substrate on which insects are raised and by the insect species. Lipid content in BSF last instar larvae and prepupae can reach 15–49% of the total dry weight (Müller et al. 2017) and the composition of the rearing substrate has a significant impact on the lipid content of BSFL (Henry et al. 2015; Leong et al. 2016; Spranghers et al. 2017; Scala et al. 2020). For example, larvae fed on chicken manure have 15–25% of lipids, 42–49% if reared on oil rich food waste, 35% on cattle manure, and 28% on pig manure (Makkar et al. 2014). The lipid fraction of BSFL is a mix of both saturated (SFAs) and unsaturated fatty acids (FAs) (UFAs) (Müller et al. 2017; Ushakova et al. 2016) and the most abundant FAs are lauric, myristic acid, palmitic, and oleic acids, regardless of feed substrate. This composition, in terms of FAs and derivatives, is favorable mainly for detergent and soap production (Franco et al. 2022a). The concentration of medium chain SFAs (lauric acid and palmitic acid) (67% of total FAs) in the BSF prepupae crude fat was higher than soybean (11% of total FAs) and palm oil (37% of total FAs) (Caligiani et al. 2018; Kroeckel et al. 2012; Li et al. 2011; Makkar et al. 2014; Ushakova et al. 2016). Furthermore, the proportion of UFAs (28% of total FAs) was lower than palm oil and soybean oil (Surendra et al. 2016). Lauric acid, which is absolutely the most prevalent FA in the lipid fraction of BSFL (Caligiani et al. 2018; Leong et al. 2015; Müller et al. 2017; Spranghers et al. 2017), is transformed into monolaurin by the reaction of glycerol and lauric acid, followed by esterification. Monolaurin is a glyceride that has antiviral, antibacterial, and antiprotozoal properties (Diclaro and Kaufman 2009). In comparison to other insects, BSFL seem not to accumulate pesticides or mycotoxins and have a higher saturated fat content, implying that the use for feed is safe and not expensive (Purschke et al. 2017). However, as for other animal species, to some extent also in BSFL, the FA profile is also affected by the feeding substrate, and this can affect the antimicrobial activity of BSFL lipids. With this research, we aim to upgrade the knowledge on the antimicrobial activity of BSFL lipids and their potential as future therapeutics. To achieve this, we evaluated for the first time the antimicrobial activity of insect crude fat deriving from BSFL reared on different substrates (Gainesville (standard diet), strawberry, orange, tangerine, and fresh dairy manure) against *Micrococcus flavus* and *Escherichia coli* cultures, as Gram-positive and Gram-negative reference strains, respectively, often used to test bioactive compound with antibacterial activity (Kumar et al. 2019). We obtained evidence for antibacterial activity in lipids extracted from larvae fed on strawberry, tangerine, and fresh dairy manure. Moreover, we analyzed the possible relation between the substrate and the larval FA profile that were not related, except for C18:0 and C18:2 CIS n6 (LA).

## Material and methods

### Insect breeding

Larval stages of BSF were provided by Xflies s.r.l. (Potenza). Ten thousand larvae were reared in an environmental chamber at 27.0 ± 1.0 °C, 70.0% relative humidity (RH). BSFL were reared on 7 kg of five different substrates (Scieuzo et al. 2022; Franco et al. 2022b); three substrates composed by 100% of agri-food chain by-products (oranges, O; tangerines, T; strawberries, S) provided by Apofruit Italia (Scanzano Jonico, Matera, Italy), fresh dairy manure (FDM) collected at “Azienda Tamburrino Mariano” (Oppido Lucano, Potenza, Italy), and a control diet composed of Gainesville diet (GD, Hogsette et al*.* 1992) is composed of 30% alfalfa, 50% wheat bran, and 20% cornmeal. At the end of the bioconversion process, whose duration depended on the feeding substrate, larvae were separated from frass, washed by distilled water and ethanol, and freeze-died at -20 °C for 24 h.

### Lipid extraction

The substrate and larval lipid extraction was carried out by Soxhlet extractor (Merck Millipore, Burlington, MA, USA). Both samples were dried at 55 °C for 48 h and finally grinded to obtain a powder form. Each replicate was composed of 7 g of sample powder. Soxhlet extraction was carried out with 150 ml of petroleum ether (according to “ILIADe 179:2018 | CLEN Method—Determination of Total Fat Content in Food Products”) and the extraction was stopped approximately after 20 extraction cycles, when the solvent, after passing in the siphon, became from light yellowish to colorless. At the end of the extraction, a mix of solvent and ether extracts was obtained. The solvent was evaporated to dryness at 65 °C using a rotating evaporator for 45 min. Then, the flask containing the extracted lipids, previously weighed as empty, was re-weighed to determine the amount of extracted lipids and the lipid yield of the samples. Extracted yield in percentage was calculated using the following formula:

$$\mathrm{Lipid }\mathrm{yield\ (\%)\ = }\frac{\mathrm{Flask }\mathrm{weight\ with\ solvent\ and\ lipids }\mathrm{(g)}\mathrm{-Empty\ flask\ weight }}{\mathrm{Dry\ weight\ of\ the\ original\ raw\ insect\ sample\ (g)}}\times 100$$

### Fatty acid profile

To perform a FA transmethylation analysis of substrate and larval extracts, a base-catalyzed procedure reported by Christie (1982) and modified by Chouinard et al. (1999) was used. The methyl esters were quantified by gas chromatograph (Agilent technologies, model 5890) fitted with an SP-2560 fused silica capillary column (100 m × 0.25 mm i.d. × 0.2 µm film thickness, Supelco, Inc., Bellefonte, PA, USA). The carrier gas, helium, was set at a constant pressure of 180 kPa, splitting flow of 50 mL/min, and injection volume of 1 µl. In column parameters, the initial temperature of the column was maintained at 170 °C for 15 min; then, with an increase of 5 °C/min, it was brought up to 240 °C. The total execution time was 64 min. By comparing the retention times of commercial standard containing 37 methyl esters of FAs (Merck Millipore, Burlington, MA, USA), FA peaks were identified. The retention times of the CLA isomers were controlled by the elution of commercial standards (Larodan AB-SE-171 65 Solna) of these FAs. The area of each individual FA identified in the sample was quantified by percentage calculation on the total area of the eluted peaks.

### Antimicrobial assays

A colony of *Escherichia coli* (Gram-negative, LMG:2092 strain) and a colony of *Micrococcus flavus* (Gram-positive, DSM 19079) were inoculated into 10 ml of sterile Luria–Bertani (LB) culture medium, prepared with 1% of tryptone (Merck Millipore, Burlington, MA, USA), 0.5% of yeast extract (Merck Millipore, Burlington, MA, USA), and 0.5% of sodium chloride (Merck Millipore, Burlington, MA, USA), and were placed in a water bath shaker at 37 °C and 150 rpm for 18 h. Agar diffusion test was carried out for evaluating the antimicrobial activity of the different lipid samples. The bacterial culture was used at a concentration of 0.6 CFU/ml for both species. Bacteria were homogeneously distributed using a cotton swab on the top of LB-Agar medium (LB with 1.5% bacteriological Agar (Merck Millipore, Burlington, MA, USA)) in sterile Petri dishes. Subsequently, 5 µl of each lipid sample was spotted onto the LB agar plates and incubated at 37 °C for 24 h. For each plate, 5 µl ampicillin at 5 mg/ml concentration was used as positive control. Each experiment was carried out in triplicate. Inhibition diameters were evaluated by AutoCAD software.

### Statistical analysis

Differences in lipid yield, FA composition, and the diameters of inhibition were analyzed by one-way Analysis of Variance (ANOVA) according to the model: Yij = m + Si + eij where Y is the single observation, m the general mean, S the effect of the growing substrate, and e the error.

Bonferroni post hoc test was used to compare the differences among means. The statistical analysis was performed through GraphPad Prism 6.0 software (GraphPad Software, Inc., La Jolla, USA). Results are presented as the mean ± standard error (SE) of three independent replicates for lipid yield and diameters of inhibition, while they are presented as the mean and standard error of the mean (SEM) of three replicates for FA analysis.

Correlation and percentage of correlation of substrate and larval FAs was performed using the “Correlation” function in excel software. The evaluation was carried out exclusively for the FAs present in all the substrates or at least on four substrates out of five.

## Results

### Lipid extraction

The proximate lipid content of BSFL and substrates (GD, Gainesville diet; T, tangerine; S, strawberry; O, orange; FDM, fresh dairy manure) was determined on a dry weight basis by Soxhlet extraction method (Table 1). Differences were identified among all the initial substrate samples, except between tangerine and strawberry samples. Focusing the attention on the different substrates on which BSFL were reared on, differences were detected among larval samples reared on fresh dairy manure and all the other substrates.

**Table 1:** Lipid content (%) of initial substrates (*GD* Gainesville diet, *T* tangerine, *S* strawberry, *O* orange, *FDM* fresh dairy manure) and of BSF larvae fed on different substrates

Sample	Initial substrates lipids (% on dry basis) (Mean ± SE)	BSFL lipids (% on dry basis) (Mean ± SE)
GD	5.08 ± 0.15^a^	22.99 ± 0.30^a^
T	0.34 ± 0.02^c^	21.89 ± 0.60^a^
S	0.38 ± 0.025^c^	20.90 ± 1.40^a^
O	0.24 ± 0.023^d^	23.00 ± 1.48^a^
FDM	3.96 ± 0.06^b^	14.29 ± 0.28^b^

Lipid content of initial substrates and BSF larvae was measured by Soxhlet extraction method. Data are presented as mean ± SE (*n* = 3). Letters indicate significant differences among all the samples (*p* < 0.01)

### Fatty acid profile

The FA profiles of the initial substrates are reported in Table 2. The FA profile was composed from 56 to 12.5% of SFAs, from 24.5 to 15% of monounsaturated FAs (MUFAs), from 64 to 29% of polyunsaturated FAs (PUFAs), from 42 to 15% of *n* − 6, and from 31 to 1.7% of *n* − 3, depending on the substrate. In all samples, except for tangerine, the most detected PUFAs was C18:2 CIS n6 (LA) (linoleic acid); the highest percentage of linoleic acid was observed in the substrate composed by Gainesville diet (almost 42%) while the lowest in tangerine substrate (almost 15%). Linoleic acid was followed by C18:3 n3 (ALA) (α-linolenic acid), except for fresh dairy manure, C18:1 CIS9 (oleic acid), and C16:0 (palmitic acid).

**Table 2:** FAs composition (%) of initial substrates (*GD* Gainesville diet, *T* tangerine, *S* strawberry, *O* orange, *FDM* fresh dairy manure)

Fatty acids	GD	S	T	O	FDM	SEM	*p* value
C4:0	0	0	0	0	7.09	-	-
C12:0	0.28^a^	0.12^c^	0.78^a^	0.40^a^	0.37^a^	0.17	< 0.0001
C14:0	0.50^d^	0.37^d^	2.37^b^	0.76^c^	2.73^a^	0.80	< 0.0001
C14:1	0	0.03^b^	0	0	2.85^a^	1.42	0.0471
C15:0	0	0.13^b^	0.61^ab^	0	1.45^a^	0.50	0.0110
C16:0	15.47^c^	8.43^d^	19.23^b^	22.85^a^	19.51^b^	3.93	< 0.0001
C16:1	0.61^d^	0.67^d^	1.97^a^	2.05^a^	1.57^c^	0.50	< 0.0001
C17:0	0	0.11^b^	0.12^b^	0.83^a^	0.89^a^	0.31	0.0004
C18:0	9.52^b^	1.76^d^	4.92^c^	2.91^d^	16.31^a^	4.24	< 0.0001
C18:1 CIS9	14.92^c^	22.37^a^	19.76^b^	13.06^d^	18.1^b^	2.65	< 0.0001
C18:2 CIS n6 (LA)	42.09^a^	35.05^b^	15.03^d^	32.28^b^	19.26^c^	8.03	< 0.0001
C18:3 n3 (ALA)	14.19^c^	28.86^a^	30.93^a^	23.93^b^	0.6^d^	8.89	< 0.0001
C18:3 n6	0	0	0	0	5.20	-	-
C20:0	0.46^b^	0.87^b^	0.79^b^	0	3.68^a^	1.25	< 0.0001
C20:1	0.92^a^	0.29^a^	0	0	0	0.40	0.2053
C20:3 n6	0	0	0	0	2.33	-	-
C20:4 n6 (AA)	0.01^b^	0.07^b^	0.06^b^	0	0.54^a^	0.18	< 0.0001
C20:5 n3 (EPA)	0	0.04^a^	0	0	0.18^a^	0.07	0.1290
C22:0	0.28^a^	0.43^a^	2.27^b^	0	2.94^b^	0.97	0.0002
C22:1	0.25^a^	0	0	0	0.04^b^	0.11	0.0235
C22:6 n3 (DHA)	0.004^a^	0	0	0	1.14^a^	0.44	0.1163
C24:0	0.28^c^	0.15^c^	1.12^a^	0.93^ab^	0.62^b^	0.30	0.0001
	**GD**	**S**	**T**	**O**	**FDM**	**SEM**	
SFAs	25.79^c^	12.46^d^	32.21^b^	28.68^c^	56.09^a^	11.30	< 0.0001
MUFAs	16.71^bc^	23.43^a^	21.77^ab^	15.11^c^	24.47^a^	3.03	0.0016
PUFAs	56.50^b^	64.20^a^	46.02^c^	56.21^b^	28.96^d^	9.69	< 0.0001
*n* − 6	42.11^a^	35.30^b^	15.09^e^	32.28^c^	27.23^d^	7.22	< 0.0001
*n* − 3	14.39^b^	28.90^a^	30.93^a^	23.93^b^	1.73^c^	8.57	< 0.0001

FA composition was evaluated by gas chromatography. Data are presented as mean of three biological replicates ± SEM and analyzed by one-way ANOVA and Tuckeypost-hoc test. For C14:1, C20:1, C20:5 n3 (EPA), C22:1 and C22:6, statistical analysis was performed with unpaired *t*-test with Welch’s correction. Different letters indicate significant differences among groups (*P* < 0.01)

The FA profiles of the larvae are reported in Table 3. For BSFL reared on the examined substrates the FA profile was composed from 71 to 79% of SFAs, from 12 to 19% of MUFAs, from 7 to 11% of PUFAs, from 5 to 9% of *n* − 6, and from 2 to 3.5% of *n* − 3, depending on the substrate where they fed on. In all samples, the most detected FAs was C12:0 (lauric acid); the highest percentage of lauric acid was observed in BSFL reared on orange substrate (almost 44%), while the lowest in BSFL reared on strawberry substrate (38%). Lauric acid was followed by C16:0 (palmitic acid), C18:1 CIS9 (oleic acid), and C18:2 CIS n6 (linoleic acid).

**Table 3:** FA composition (%) of BSFL fed on different substrates (*GD* Gainesville diet, *T* tangerine, *S* strawberry, *O* orange, *FDM* fresh dairy manure)

Fatty acids	GD	S	T	O	FDM	SEM	*p* value
C4:0	3.36^b^	6.87^ab^	5.11^ab^	3.10^b^	8.95^a^	1.58	0.0040
C10:0	0.75^b^	0.26^c^	0.33^bc^	0.53^bc^	1.29^a^	0.24	< 0.0001
C12:0	37.63^b^	38.00^b^	40.38^ab^	43.93^a^	39.57^ab^	1.67	0.0095
C14:0	8.42^ab^	8.68^ab^	9.67^a^	9.11^ab^	7.30^b^	0.59	0.0136
C14:1	0.29^a^	0.08^a^	0.23^a^	0.23^a^	0.08^a^	0.07	0.0355
C15:0	0.21^ab^	0.25^a^	0.26^a^	0.32^a^	0.02^b^	0.07	0.0042
C16:0	16.74^a^	14.31^a^	16.08^a^	15.39^a^	14.71^a^	0.77	0.1017
C16:1	0.40^b^	3.27^a^	0.07^b^	0.13^b^	2.25^a^	0.85	< 0.0001
C17:0	3.64^a^	0	3.78^a^	3.74^a^	1.43^b^	0.60	< 0.0001
C17:1	0.30^a^	0	0.29^a^	0.43^a^	0.31^a^	0.07	0.0157
C18:0	3.02^a^	2.71^a^	2.87^a^	2.75^a^	3.14^a^	0.22	0.6418
C18:1 CIS6	0.23^b^	0.36^ab^	0.14^ab^	0.20^b^	0.40^a^	0.08	0.0224
C18:1 trans 11 (TVA)	0.44^a^	0.24^a^	0	0.15^a^	0.24^a^	0.08	0.2265
C18:1 CIS9	12.31^a^	12.25^a^	11.53^a^	11.25^a^	10.11^a^	0.77	0.2109
C18:1 CIS11	0.05^a^	0.12^a^	0.05^a^	0.15^a^	0.07^a^	0.06	0.7528
C18:2 CIS n6 (LA)	8.38^a^	6.98^a^	4.48^a^	5.57^a^	5.41^a^	1.12	0.0516
C18:3 n3 (ALA)	1.86^b^	2.53^ab^	2.11^ab^	1.20^b^	3.45^a^	0.54	0.0044
C20:0	0.14^a^	0	0	0	0.17^a^	0.03	< 0.0001
C20:1	0.11^a^	0	2.12^a^	0.16^a^	0.02^a^	0.06	0.0912
C22:0	0.64^a^	0.86^a^	0.74^a^	0.41^a^	0.97^a^	0.19	0.1012
C22:1	0.15^a^	0	0.13^a^	0.10^a^	0.05^a^	0.04	0.0930
	**GD**	**S**	**T**	**O**	**FDM**	**SEM**	
SFAs	74.53^ab^	71.93^b^	79.22^a^	79.29^a^	77.63^ab^	2.10	0.0135
MUFAs	14.28^a^	16.31^a^	12.63^a^	12.80^a^	13.50^a^	1.20	0.1291
PUFAs	10.87^a^	10.37^a^	7.43^a^	7.17^a^	8.86^a^	1.26	0.0727
*n* − 6	9.01^a^	7.84^a^	5.31^a^	5.97^a^	5.41^a^	1.16	0.0333
*n* − 3	1.86^a^	2.53^ab^	2.12^a^	1.20^ab^	3.45^a^	0.54	0.0044

FA composition was evaluated by gas chromatography. Data are presented as mean of three biological replicates ± SEM and analyzed by one-way ANOVA and Tuckey post-hoc test. For C20:0 statistical analysis was performed with unpaired t-test with Welch’s correction. Different letters indicate significant differences among groups (*p* < 0.01)

**Fig. 1:**
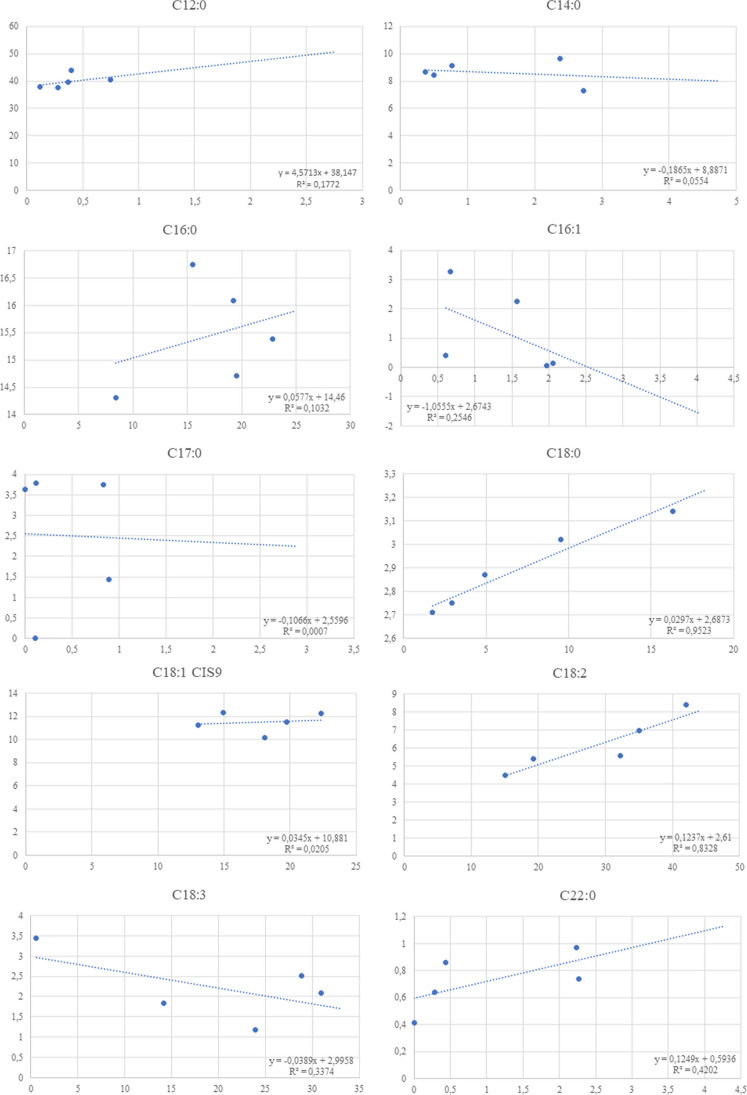
Correlation between FAs of the substrate and the larvae. X axis report % on FA in substrates, Y axis report % on FA in larvae. Data are presented as mean of three biological replicates. *p* value: C12 = 0.4803; C14 = 0.7032; C16 = 0.5982; C16:1 = 0.3860; C17 = 0.9660; C18 = 0.0045; C18:1 CIS9 = 0.8181; C18:2 = 0.0306; C18:3 n3 (ALA) = 0.3045; C22 = 0.2368

Correlation between FAs of substrates and larvae is reported in Fig. 1. It was possible to evaluate the possible correlation exclusively for the FAs present in all the substrates or at least on four substrates out of five. A linear correlation was highlighted for C18:0 and C18:2 CIS n6 (LA), with 97.58% and 91.26% of correlation. A possible correlation could be reported also for C12:0, but an anomalous observation did not allow to confirm a direct correlation between this FA in substrates and in larvae.

### Antimicrobial assay

**Fig. 2:**
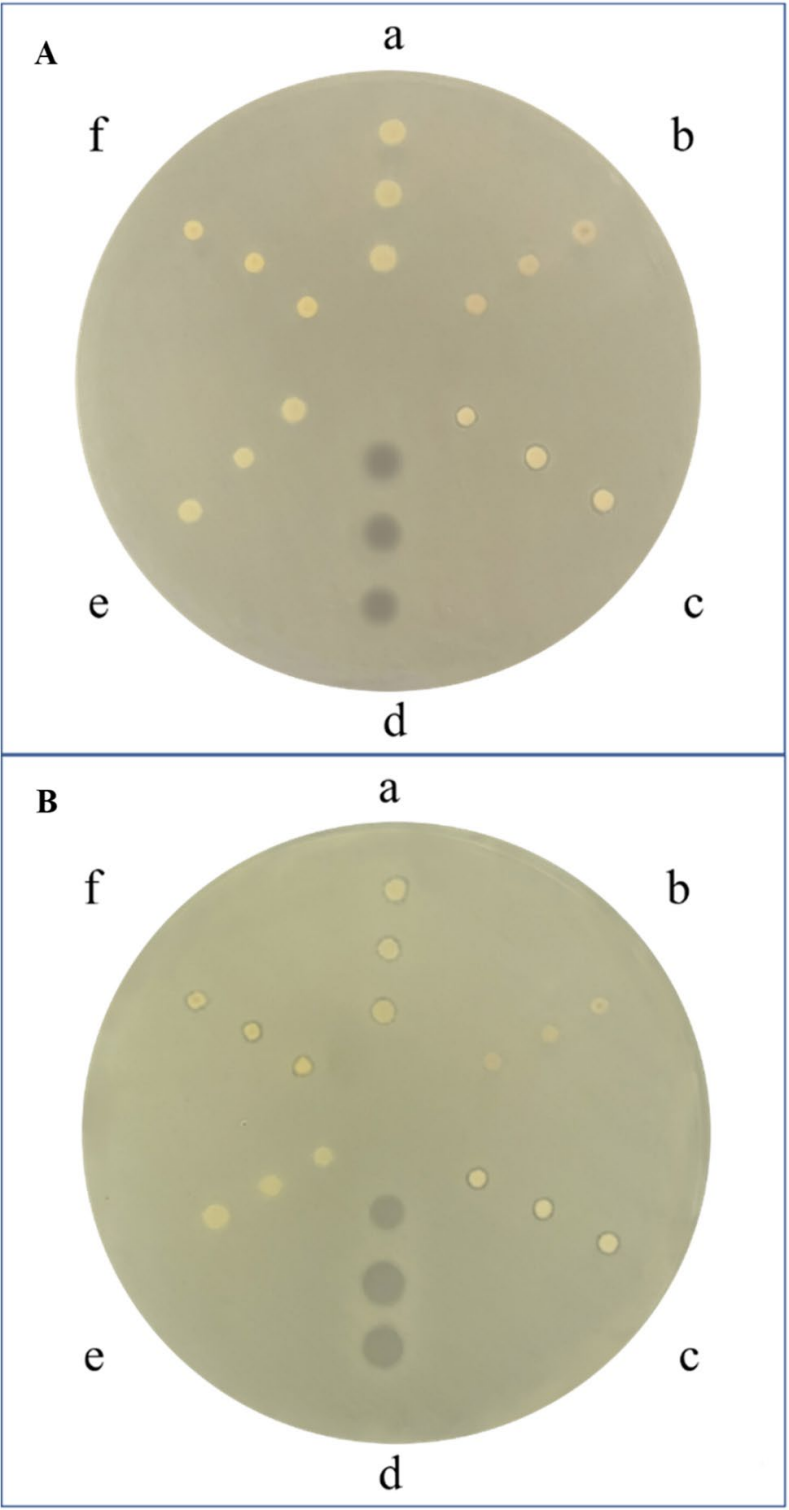
Microbiological assay and the growth inhibition zone developed around the crude fat extracted from BSFL fed on different substrates ((a) strawberry, (b) orange, (c) fresh dairy manure, (d) AMP, (e) Gainesville diet, f tangerine) against *Escherichia coli* in **A** and *Micrococcus flavus* in **B**

The potential antimicrobial activity of BSFL lipids was assessed through agar diffusion tests. *M. flavus* was chosen as a representative strain of Gram-positive bacteria, while *E. coli* as Gram-negative one, both often used to test bioactive compounds with antibacterial activity (Kumar et al. 2019). Ampicillin was used as a positive control. The agar diffusion test did not highlight any significant antimicrobial activity of the samples against *E. coli* except for lipids extracted from larvae reared on fresh dairy manure (Fig. 2A). *M. flavus* showed a sensitivity towards BSFL lipids extracted by larvae reared on strawberry, tangerine, and fresh dairy manure with growth inhibition zones ranging from 1.38 to 1.51 mm, respectively (Fig. 2B, Table 4).

**Table 4:** Diameters of inhibition of lipids (mm) resulted active, as reported in Fig. 1

	A – c	B – a	B – c	B – f
Diameter of inhibition (mm)	1.17 ± 0.06^b^	1.38 ± 0.04^ab^	1.48 ± 0.02^a^	1.51 ± 0.09^a^

Data are presented as mean of three biological replicates + SE. Three biological replicates. Different letters indicate significant differences among groups (*p* = 0.0313). A – c = fresh dairy manure against *E. coli*; B – a = strawberry against *M. flavus*; B – c = fresh dairy manure against *M. flavus*; B – f = tangerine against *M. flavus*

## Discussion

The aim of this study is to deepen the understanding of the influence of the larval feeding substrate on lipid content, FA profile, and antimicrobial activity of BSFL lipids. The quantity and quality of FAs in BSFL can differ with the progression of the larval development and the use of different growing substrates (Li et al. 2022). For this reason, it is plausible to hypothesize that both lipid content and FA profile of the feeding substrate could influence the quantity and quality of fat in larvae.

Generally, the amount of unsaturated FAs in insects is raised by the high level of unsaturated FAs in the substrate, even if they can accumulate just some of them, such as oleic acid, linoleic acid, and α-linolenic acid (Hoc et al. 2020). Indeed, by feeding insects with substrate improved by fatty source, the amount of linoleic and α-linolenic acid could increase (Oonincx et al. 2019). However, Meneguz et al. (2018) assert that additional nutrients, such as carbohydrates, have a major impact on the FA content of BSFL compared to the FA composition of the substrate. Recently, two genes coding for acetyl-CoA carboxylase and FA synthase were characterized, confirming that larvae can de novo synthesize some FAs, starting from carbohydrates of their diet (Giannetto et al. 2020).

In our study, on all substrates, the crude fat extracted from the larvae contained large concentrations of SFA, among which, according also to literature data, lauric acid was the most abundant, followed by myristic, palmitic, and oleic acids, regardless of feed substrate. Spranghers et al. (2017), indeed, highlighted that the lipids of BSF prepupae were predominantly made up of lauric acid, even when the BSFL were fed on substrate with a very low amount of the aforementioned acid (Spranghers et al. 2017). They suggested that the FA content of the rearing feeding substrate has no direct effect on the larval FA composition, which was influenced more by carbohydrates (Oonincx et al. 2019; Spranghers et al. 2017). Also in our study, the experimental comparison between FA profiles of larvae and feeding substrates does not display a correlation between these macro categories. Indeed, our results on the FA analysis, in line with several studies (Oonincx et al. 2019; Meneguz et al. 2018; Spranghers et al. 2017), showed that larval FAs typically did not follow the same trend of the FAs contained in feeding substrates, except for stearic and linoleic acids, for which a linear correlation was detected. For the analyzed substrates starting from a very low amount of lauric or myristic acid in the feeding substrates, BSFL were able to synthesize them confirming that the portion of SFAs contained in the FA profile is not influenced by the feeding substrate (Hoc et al. 2020). Due to the FA metabolism in BSF, it was noted that there was only a restricted chance to alter the FA composition of BSFL by the substrate (Oonincx et al. 2019). Since some FAs may be produced or transformed by insects in other FAs, it is unlikely to find the same FAs acid in insects and substrates and the strict comparison between them is a too simplistic approach (Hoc et al. 2020). BSF larvae can also synthesize palmitic (C16:0) and stearic acid (C18:0), as demonstrated by Hoc et al. (2020) in which despite high concentrations of palmitic acid in administered diet, larvae biosynthesized around 50% of FA present in their body rather than a simple accumulation (Hoc et al. 2020). Another example of differences in FA concentration is linked to the lowest content of α-linolenic acid (18:3n-3) and *n* − 3 found in fresh dairy manure and the higher value in larvae fed with this substrate. The amount of linoleic (C18:2 n6) and α-linolenic (C18:3 n3) acids differs even in insects raised on comparable control substrates (Oonincx et al. 2019; Danieli et al. 2019; Spranghers et al. 2017). This suggests that other factors, such as the environmental rearing conditions, Gene-by-Environment (GxE) interactions, and inoculation of some *Lactobacillus* strains in the feeding substrate (Somroo et al. 2019; Greenwood et al. 2021), influence the quantity of essential FAs in insects in addition to the used substrate (Riekkinen et al. 2022). Summarizing, although several studies show how the substrate influences the different nutritional components of the larvae, the direct correlation between a specific component present in the substrate and the corresponding element in the larvae is not clear and cannot be generalized. Indeed, while opposing data are reported for BSF, in other species, the FA profile appears to be constant and strictly substrate dependent/independent: in *Tenebrio molitor*, for example, FA profile is independent from the substrate, while in *Acheta domesticus* is directly influenced (Riekkinen et al. 2022).

Concerning the microbiological assay, our preliminary study showed, although faint, an antimicrobial activity of crude fat extracted from BSF. It is reasonable to attribute the observed activity exclusively to BSFL lipids, since the extraction methodology applied allows the isolation of the sole lipid content. In particular, BSFL crude fat was highly selective for the Gram-positive *M. flavus*. In line with the current literature, Gram-positive bacteria with the single thick peptidoglycan layer are more sensitive to FAs then Gram-negative species, allowing intermediate- and long-chain FAs to penetrate and exert their subsequent toxic action (Saviane et al. 2021; Koutsos et al. 2022). Substrate influences the specific FA content, and these findings could be transferred to antimicrobial results, in which BSFL reared on different substrates showed different results, depending also by bacterial species tested. These findings indicate that the administration of different by-products deriving from agrifood or zootechnical chains can affect the antimicrobial activity of BSFL lipids. Specifically, we detected differences for C4:0 (butyric acid), C10:0 (capric acid), C16:0 (palmitoleic acid), C18:3 n3 (ALA) (α-linolenic acid), and C20:1 (eicosenoic acid). According to other researchers, all of these FAs showed antimicrobial activity (Van Immerseel et al. 2005; Sun et al. 2019; Cox et al. 1994; Namkung et al. 2011; Wille and Kydonieus 2003; Kabara et al. 1972; Huang et al. 2010; McGaw et al. 2002; Sado-Kamdem et al. 2009; Elshobary et al. 2020; Ohta et al. 1995; Shilling et al. 2013; Huang et al. 2014). Intersecting results of FA components (Table 3) and of antimicrobial assay (Fig. 2), we highlighted the differences of FAs among samples that might confer antibacterial activity; starting from this, we can hypothesize that these differences among specific FAs could improve or determine the antimicrobial activity*.*

In detail, capric acid (C10:0) detected in FA profile of larvae reared on fresh dairy manure showed statistical difference with all the BSFL samples. According to Shilling et al. (2013), capric acid induces an inhibition of *Clostridium difficilis* (Gram-positive) growth (Shilling et al. 2013), while Huang et al. (2014) found that it showed activity against *Propionibacterium acnes* (Gram-positive) (Huang et al. 2014). Moreover, it shows activity against several other bacterial strains (Sprong et al. 2001), both Gram-positive and Gram-negative bacteria (Desbois and Smith 2010).

Butyrate (C4:0) can exert multifaceted antibacterial and immune-modulatory effects against bacteria in vitro and in vivo. A study assessed the minimal inhibitory concentrations (MICs) of direct exposure of butyrate against various Gram-positive and Gram-negative bacteria revealing a bacterial inactivation with a MIC between 11 and 21 mmol/L (Du et al. 2021). The butyrate exposure clearly revealed a membrane damage with depolarization and leakage of intracellular electrolytes. Given the ability of butyrate to trigger host defense peptides in vitro and in in vivo animal feeding trials, it is becoming increasingly in use as a feed additive to protect from bacterial infections (Du et al. 2021). According to Van Immerseel et al. (2005), butyric acid reduces cecal colonization immediately after infection, fecal shedding, and, as a result, reduces environmental contamination caused by *Salmonella enteritidis*-infected broilers (Van Immerseel et al. 2005). Moreover, *Salmonella* spp. colonization in the ceca (Cox et al. 1994) and *Salmonella enteritidis* penetration in chicken cecal epithelial cells (Van Immerseel et al. 2005) are also reduced.

α-linolenic acid (C18:3 n3) showed an effective antibacterial activity against methicillin-resistant *Staphylococcus aureus* (Ohta et al. 1995; Sado-Kamdem et al. 2009). In lipids extracted by BSFL reared on tangerine substrate, eicosenoic acid (C20:1) showed statistical differences with all the BSFL samples analyzed; the amount of this FA is higher in larvae reared on tangerine substrate than all the others larval samples, with an antibacterial effect on *M. flavus*.

As reported by Mohy El-Din and El-Ahwany (2016), eicosenoic acid has antibacterial properties due to its capacity to lyse bacterial protoplasts (Mohy El-Din and El-Ahwany 2016).

About the antimicrobial activity shown by lipids deriving from larvae reared on strawberry substrate, palmitoleic acid (C16:1) showed a higher amount and a statistical difference with the respective FA from larvae reared on Gainesville diet and orange substrate, which showed no activity against *M. flavus.* In this case, as reported by Wille and Kydonieus, palmitoleic acid showed antibacterial activity against Gram-positive cocci (*Pneumococcus*, *Streptococcus*, *Micrococcus*, *Staphylococcus*) (Wille and Kydonieus 2003) and against *Corynebacterium*, *Nocardia asteroides*, and *Candida albicans* (Kabara et al. 1972). Specifically, in this research, antimicrobial effects were recorded by palmitoleic isomer isolated from human sebum. Moreover, Huang et al. found a 100% inhibition against oral pathogens such as *Candida albicans*, *Streptococcus mutans*, *Porphyromonas gingivalis*, *Aggregatibacter actinomycetemcomitans*, and *Fusobacterium nucleatum* (Huang et al. 2010).

Considering the limited numbers of studies and reviewed publications regarding the antimicrobial effects of BSFL extracted crude fats, distinct conclusions should be interpreted with attention, and it is necessary to explore the complexity of antimicrobial activity exerted in well-designed studies in the future. Although only a few in vitro studies revealed direct antibacterial effects of BSFL crude fat, further studies should therefore expand our preliminary findings. Moreover, since the substrates that showed more antimicrobial activity against *M. flavus* are the ones composed by strawberry and tangerine, it could be interesting to use these two different ingredients mixed together to improve the antimicrobial ability of BSFL FA profile, in order to use the extracted lipids in different industrial field such as cosmetic and feed industry.

The data in the current literature are supported by our results, demonstrating an effective antimicrobial inhibition against several strains, provided by these insects FAs. These findings demonstrate that BSFL FA profile could be modified through different diet administration, although the missing relation between FAs of the substrate and larvae implies that the real challenge in order to modulate and use them in the most appropriate way is the identification of biochemical pathways for FA synthesis by BSF. Indeed, the synthesis of these FAs is also influenced by various factors, such as other nutrients, mainly carbohydrates, larval development stages, and rearing conditions, such as optimal temperature. Understanding these factors can help in optimizing the production of lipids for various applications, including as new antibacterial agents.

## Data Availability

The datasets used and/or analyzed during the current study are available from the corresponding author on reasonable request.
